# Successful pregnancy following IVF in a woman with hypopituitarism secondary to germinoma and thrombophilia: a case report and literature review

**DOI:** 10.5935/1518-0557.20260003

**Published:** 2026

**Authors:** Teresa Gastañaga-Holguera, Vanesa Rayo-López, Isabel Campo-Gesto, Marta Calvo-Urrutia

**Affiliations:** 1 Assisted Reproduction Unit. Hospital Clinico San Carlos. Complutente University of Madrid. Madrid, Spain

**Keywords:** hypopituitarism, pituitary germinoma, IVF, assisted reproductive technology, thrombophilia, pregnancy

## Abstract

A 32-year-old woman with panhypopituitarism following surgical resection, chemotherapy, and radiotherapy for a pituitary germinoma underwent assisted reproductive treatment. Management involved comprehensive hormone replacement therapy, ovulation induction with exogenous gonadotropins, in vitro fertilization (IVF), and thromboprophylaxis with low molecular weight heparin due to a diagnosed thrombophilia. A fresh embryo transfer resulted in a successful singleton pregnancy. The pregnancy progressed uneventfully and reached full term and a healthy neonate was delivered. This case highlights the feasibility of pregnancy in women with complex endocrine and thrombotic conditions and adds to the limited literature supporting IVF in women with significant hypothalamic-pituitary axis compromise.

## INTRODUCTION

Hypopituitarism is characterized by a partial or complete deficiency of anterior pituitary hormones, with etiologies ranging from congenital defects to acquired conditions, including pituitary tumors (Wang *et al*., 2018). Among these, germinomas represent a rare subtype, accounting for approximately 3% of all primary brain tumors, and primarily affect children and young adults (Kong *et al*., 2018; Hayashi *et al*., 2017). Diagnosis relies on histopathological examination due to the often nonspecific nature of the symptoms. These tumors require multimodal treatment, typically including transsphenoidal resection, cisplatin-based chemotherapy regimens, and radiotherapy, which frequently results in irreversible pituitary dysfunction (Tsoukalas *et al*., 2013).

Hormone replacement therapy is essential for managing hypopituitarism-related symptoms and preventing complications such as osteoporosis, adrenal insufficiency, and infertility. In addition, the use of assisted reproductive technologies (ART) becomes particularly challenging due to gonadal dysfunction in women exposed to gonadotoxic therapies, or in those with additional prothrombotic conditions (Chen *et al*., 2024; Wang *et al*., 2018).

Fertility preservation strategies, including gamete cryopreservation and ovarian cortex preservation, are currently recommended for patients scheduled to receive potentially gonadotoxic treatments. The extent of gonadal damage largely depends on the specific chemotherapy regimens employed, with a significant risk of premature ovarian failure. Even when pituitary radiation is precisely targeted, it may disrupt the reproductive neuroendocrine axis (Hayashi *et al*., 2017). Consequently, female cancer survivors treated with systemic chemotherapy or radiotherapy exhibit lower pregnancy and live birth rates compared to reproductive-aged women without relevant medical histories undergoing ART. Notably, patients undergoing bone marrow or hematopoietic stem cell transplantation represent the highest-risk group for ovarian failure, with incidence rates reaching up to 90% (Barton *et al*., 2012).

Currently, there is limited documented evidence of successful pregnancies in women with hypopituitarism who have undergone chemotherapy or radiotherapy. In such cases, appropriate hormone replacement therapy, designed to closely mimic the physiological hormonal fluctuations of pregnancy, is critical for successful outcomes (Wang *et al*., 2018). Here, we present a rare case of a successful pregnancy in a woman with hypopituitarism due to a pituitary germinoma, compounded by thrombotic risk and prior exposure to chemoradiotherapy.

## CASE REPORT

A 32-year-old woman with a history of pituitary germinoma diagnosed at age 15 presented for fertility evaluation. Initial symptoms before tumor diagnosis included amenorrhea, polyuria-polydipsia, visual field defects, and slowed growth. Hormonal assessment confirmed central adrenal insufficiency, central hypothyroidism, and hypogonadotropic hypogonadism. Magnetic resonance imaging (MRI) revealed a 12 mm sellar mass compressing the optic chiasm. Transsphenoidal resection was performed, and histopathology confirmed a germinoma. Postoperatively, she required hormonal replacement therapy and received three cycles of BEP chemotherapy (bleomycin, etoposide, cisplatin) and cranial radiotherapy. During the first chemotherapy cycle, she presented with left hemiparesis and generalized seizures, due to superior sagittal sinus thrombosis requiring anticoagulation. At the conclusion of chemotherapy, the patient developed an eating disorder necessitating inpatient treatment, showing a favorable clinical course.

At age 17, a second thrombotic event occurred. Thrombophilia testing revealed heterozygosity for the MTHFR mutation in the MTHFR gene and elevated Factor VIII (208.1%), contributing to thrombophilia. The patient initiated growth hormone therapy due to asthenia, loss of muscle mass, and severe osteoporosis (T-score: -3.3 in the lumbar spine, -2.4 in the hip) at the age of 23.

At 32 years of age, she expressed the desire to conceive as a single-parent. Her ongoing hormone replacement regimen included hydrocortisone 10 mg every 12 hours, subcutaneous growth hormone at 0.6 mg daily, levothyroxine 100 µg/day, and combined oral contraception. Initial fertility evaluation revealed a baseline transvaginal ultrasound showing a linear endometrium measuring 2 mm, 3 antral follicles in the right ovary, and 2 in the left. Serum Anti-Müllerian Hormone (AMH) level was 1.14 ng/mL.

The patient had not previously attempted to conceive and underwent four intrauterine insemination (IUI) cycles with donor sperm using recombinant follicle stimulation hormone (FSH) and luteal hormone (LH), resulting in a single biochemical pregnancy. Ovarian responsiveness to low-dose exogenous gonadotropins was suboptimal, characterized by prolonged stimulation protocols and one cycle cancellation due to an inadequate response. Consequently, in vitro fertilization (IVF) was initiated using combined recombinant FSH (rFSH) and (rLH) (Pergoveris®, Merck) at a dose of 250 IU/day. On stimulation day 13, two dominant follicles (19 mm and 18 mm) were visualized in the right ovary, and one 12 mm follicle in the left one, with an endometrial thickness of 7.8 mm. Final oocyte maturation was triggered with human chorionic gonadotropin (hCG) (Ovitrelle^®^, Merck Serono). Two metaphase II (MII) oocytes were retrieved, both of which developed into blastocyst stage. A day 5 fresh blastocyst, grade 5AB according to Gardner and Schoolcraft grading system was transferred, resulting in an ongoing pregnancy. The second blastocyst was cryopreserved. Luteal phase support consisted of vaginal progesterone at a dose of 400 mg every 12 hours.

Prophylactic anticoagulation with low molecular weight heparin (Innohep^®^, 10,000 IU) was maintained throughout treatment. Growth hormone therapy was discontinued, while levothyroxine dosage was adjusted to maintain optimal free thyroxine (T4) levels. Hydrocortisone dosing was progressively increased during the third trimester. The pregnancy evolved uneventfully. Labor was induced at 38 weeks of gestation using prostaglandins, and vacuum-assisted delivery (Mystic-type device) was performed during the second stage of labor. A healthy female neonate weighing 2,840 grams was delivered with Apgar scores of 9 and 10 at 1 and 5 minutes, respectively. The postpartum period was uneventful, and the patient resumed her individualized hormone replacement therapy.

## DISCUSSION

This case illustrates the feasibility of fertility treatment in a patient with complex endocrine and thrombotic history, highlighting several key considerations in managing ART in women with hypopituitarism.

### Initial diagnosis and treatment

Hypopituitarism leads to multiple hormonal deficiencies involving several endocrine axes. Administration of adequate exogenous hormone replacement is essential to replicate the physiological function of the hypothalamic-pituitary-ovarian axis. Particular attention is given to central adrenal insufficiency and hypothyroidism due to their critical systemic effects (Chen *et al*., 2024). These patients also experience hypogonadotropic hypogonadism, in which deficient secretion of FSH and LH compromises reproductive capacity. Nonetheless, the administration of exogenous gonadotropins may induce an adequate ovarian response, making pregnancy possible in selected cases (Salle *et al*., 2000).

This patient was treated with replacement therapy with hydrocortisone, growth hormone (GH), levothyroxine, and combined estrogen-progestin therapy, with periodic adjustments guided by clinical and laboratory findings. Optimal hormone replacement is essential in these patients to avoid both deficiencies and excesses that may negatively impact metabolic, cardiovascular, and bone health (Chen *et al*., 2024).

GH deficiency represents another key feature of hypopituitarism and is associated with impaired body composition, reduced bone mineral density, and decreased quality of life (Wang *et al*., 2015). Severe osteoporosis (T-score: -3.3 in the spine, -2.4 in the hip) prompted the initiation of GH therapy, which led to clinical and biochemical improvement. However, GH therapy was discontinued prior to conception due to the lack of robust safety data during pregnancy. GH deficiency may also impact fertility by altering gonadotropin dynamics and follicular development (Wang *et al*., 2015). Although some evidence suggests GH supplementation may enhance follicular responsiveness in women with hypogonadotropic hypogonadism undergoing ART, its effect on pregnancy outcomes remains inconclusive (Salle *et al*., 2000). Decisions regarding GH continuation during pregnancy should be individualized (Aulinas *et al*., 2022; Vila & Fleseriu, 2020).

Hypopituitarism secondary to pituitary germinoma is rare, but often results in permanent endocrine dysfunction and significantly affects quality of life. Intracranial germinomas account for approximately 3% of primary brain tumors in Western countries, with higher incidence in children and young adults (Hayashi *et al*., 2017). Early diagnosis and appropriate treatment are critical to prevent irreversible complications and require long-term multidisciplinary follow-up (Tsoukalas *et al*., 2013). In this case, the diagnosis was established during adolescence based on clinical signs of hormonal and compressive symptoms. Surgical resection followed by adjuvant chemoradiotherapy achieved complete oncological remission but resulted in permanent panhypopituitarism, necessitating lifelong hormone replacement therapy.

The BEP regimen (bleomycin, etoposide, cisplatin) remains the standard chemotherapeutic protocol for germ cell tumors, including intracranial dysgerminoma (Vitale, 2024). While some agents, such as alkylating drugs, exhibit high gonadotoxic potential, platinum-based compounds pose a variable risk. Targeted therapies and immunotherapies appear to exert less detrimental effects on ovarian function. The degree of gonadotoxicity depends on factors such as patient age, pre-treatment ovarian reserve, treatment duration, and cumulative drug exposure. Bleomycin, a glycopeptide antibiotic, is generally regarded as posing minimal gonadotoxic risk (Vitale, 2024; Satoh *et al*., 2015). Etoposide, although often classified as a topoisomerase II inhibitor, can induce follicular damage. Despite this, many patients treated with BEP retain menstrual function and achieve spontaneous pregnancies (Brewer *et al*., 1999). The impact of cisplatin on ovarian reserve remains incompletely understood, although it may reduce follicular count and steroidogenic activity. Some studies have associated both cisplatin and etoposide with premature ovarian insufficiency (Hayashi *et al*., 2017). Thus, while the BEP regimen may compromise ovarian reserve, fertility is frequently preserved (Kang *et al*., 2008; Brewer *et al*., 1999). Fertility preservation strategies should be considered before initiating treatment; however, in this case, was not performed as in that moment, those techniques were not standardized.

### Fertility evaluation and ART approach

Hypogonadotropic hypogonadism hinders pregnancy in women with hypopituitarism, as the absence of physiological pulsatile release of endogenous gonadotropins results in follicular quiescence. Consequently, pharmacological ovulation induction with exogenous gonadotropins is required, typically through ART (Wang *et al*., 2015).

Achieving pregnancy in women with panhypopituitarism presents multiple challenges, particularly in the context of prior oncologic therapies. Adequate control of central adrenal insufficiency and hypothyroidism is essential prior to attempting conception (Chen *et al*., 2024). Hypogonadotropic hypogonadism necessitates exogenous gonadotropin stimulation for follicular development, with LH supplementation often required to optimize estradiol production and oocyte maturation (Salle *et al*., 2000). Data on fertility rates in women with hypopituitarism secondary to pituitary disorders remain limited. According to a systematic review published in 2020, women with hypopituitarism receiving adequate hormone replacement therapy may conceive spontaneously, but often require ART. Women with hypopituitarism exhibit fewer ovulatory cycles, lower pregnancy rates, and higher miscarriage rates compared to women with isolated hypogonadotropic hypogonadism (Attard *et al*., 2021; Vila & Fleseriu, 2020). This is believed to result from the deficiency of additional pituitary hormones, such as growth hormone (GH), which also play a role in reproductive function. Reported fertility rates in women with hypopituitarism who underwent ART range from 47% to 76%, whereas in those with isolated hypogonadotropic hypogonadism, rates exceed 81% (Vila & Fleseriu, 2020).

Most females with hypopituitarism exhibit normal serum anti-Müllerian hormone (AMH) concentrations. However, some women with severe congenital hypopituitarism, exhibit lower serum AMH levels, suggesting that chronic gonadotropin deficiency may impair follicular maturation and development (Fitz *et al*., 2023; Deubzer *et al*., 2014). Additionally, reduced uterine size and ovarian volume have been documented in these patients, likely due to both estrogen and growth hormone deficiencies (Chen *et al*., 2024; Aulinas *et al*., 2022).

In the present case, although ovarian reserve was compromised, intrauterine insemination (IUI) was initially attempted due to the patient’s age, lack of prior spontaneous conception attempts, and the less invasive nature of IUI compared to IVF.

Despite impaired ovarian reserve and a history of thrombosis, this patient achieved a successful full-term pregnancy following individualized IVF treatment. To date, only a few cases of pregnancy following IVF in women with germinoma-induced hypopituitarism have been reported (Hayashi *et al*., 2017; Kitajima *et al*., 2003), and only one case report involving previous chemotherapy and radiotherapy for a brain tumor (Hayashi *et al*., 2017).

In patients with central hormonal deficiency, the administration of exogenous luteinizing hormone (LH) is essential for proper follicular development, as LH deficiency negatively impacts estradiol secretion and oocyte maturation. The expected duration of ovarian stimulation in women with hypopituitarism is typically longer compared to patients without this condition, likely due to prolonged ovarian suppression (Chen *et al*., 2024). In this case, ovulation was triggered after 13 days of stimulation. Luteal phase support was provided with twice-daily vaginal progesterone, following institutional protocol.

The American Society for Reproductive Medicine (ASRM), consistent with most clinical guidelines, recommends luteal phase progesterone supplementation in IVF cycles, even when ovulation is triggered with hCG. This is because hCG administration may reduce LH pulsatility, thereby impairing corpus luteum function (Garg *et al*., 2024; Van der Linden, 2015).

It is important to consider that in women with hypogonadotropic hypogonadism, the absence of endogenous estrogens may hinder proper endometrial development, potentially affecting implantation rates (Chen *et al*., 2024). In this patient, the endometrium exhibited a proliferative pattern and adequate thickness; since serum progesterone at the time of ovulation trigger was lower than 1.5 ng/mL, a fresh embryo transfer was performed. At the treating Center, clinical pregnancy and positive β-hCG rates following fresh transfer are comparable to those observed with frozen embryo transfer.

The BEP regimen poses variable gonadotoxic risks. While bleomycin is considered low-risk, cisplatin and etoposide have been implicated in ovarian damage and impaired steroidogenesis (Satoh *et al*., 2015; Kang *et al*., 2008; Brewer *et al*., 1999). Nevertheless, many women maintain menstrual function post-treatment. Fertility preservation should be addressed prior to chemotherapy initiation, although this was not feasible in the present case.

GH has been suggested to influence reproductive outcomes in hypopituitarism. Although its role in ART remains debated, some evidence suggests that GH may enhance follicular responsiveness and improve endometrial receptivity (Chen *et al*., 2024; Vila & Fleseriu, 2020). Due to limited safety data, GH was discontinued prior to embryo transfer in this case. Achieving successful pregnancies with high live birth rates remains a challenge in this patient population, as the response to ART may be compromised by the influence of multiple hormonal deficiencies on reproductive function (Chen *et al*., 2024).

### Pregnancy and delivery outcomes

Gestational complications in patients with hypopituitarism remain poorly characterized, with current knowledge primarily derived from case series (Feferkorn *et al*., 2022). An increased risk of obstetric complications has been reported. According to a 2009 study (Kübler *et al*., 2009), up to 42% of newborns from mothers with hypopituitarism were small for gestational age. This may be related to uteroplacental dysfunction, although the etiology remains unclear-whether it is due to inadequate uterine preparation or an altered maternal neuroendocrine response, and further research is needed. Hormonal deficiencies beginning in early life, as in this patient, may impair uterine and ovarian development, potentially affecting uteroplacental function and leading to adverse pregnancy outcomes, even when appropriate hormone replacement is administered during pregnancy (Vila & Fleseriu, 2020).

Women with hypopituitarism also appear to have higher rates of miscarriage, intrauterine growth restriction (IUGR), and cesarean delivery (Chen *et al*., 2024). However, no significant differences have been observed in the incidence of gestational hypertension, gestational diabetes, placental abruption, or preterm birth (Vila & Fleseriu, 2020). An increased risk of maternal infections and congenital malformations has also been noted, though these findings should be interpreted with caution due to limited sample sizes, and larger studies are needed to confirm these associations (Feferkorn *et al*., 2022). This case highlights the possibility of achieving a healthy, term pregnancy with appropriate hormonal management and coordinated multidisciplinary care.

The presence of thrombosis in the superior sagittal and sigmoid sinuses, along with recurrence of thrombotic episodes, prompted further investigation to rule out thrombophilia. Although no high-risk mutations were identified-such as Factor V Leiden or the G20210A mutation in the prothrombin gene-heterozygosity for the C677T variant in the *MTHFR* gene was detected, accompanied by markedly elevated Factor VIII levels (above 200%). While heterozygous *MTHFR* mutations are not considered to significantly increase thrombotic risk, elevated Factor VIII levels are clearly associated with an increased risk of venous thrombosis (Bardan *et al*., 2024; Lowe *et al*., 2023; Romero *et al*., 2007).

Given the patient’s history and the elevated risk of thrombotic recurrence, anticoagulation therapy with low molecular weight heparin (LMWH) was initiated throughout ART, pregnancy, and the postpartum period, each of which is recognized as a prothrombotic state. Anticoagulation management in women with prior thrombotic events and pregnancy desire is complex and requires close monitoring to balance the prevention of thromboembolic complications against the risk of bleeding-particularly during invasive procedures such as oocyte retrieval or delivery (Bardan *et al*., 2024).

According to current Spanish clinical guidelines, the use of LMWH is indicated in women with a history of venous thromboembolism and persistent risk factors. In this case, tinzaparin was administered at a daily dose of 10,000 IU.

During pregnancy, levothyroxine was titrated to maintain optimal free T4 levels. In women with central hypothyroidism, the thyroid gland remains structurally intact and may respond to hCG stimulation. This raises questions regarding thyroid hormone requirements in pregnant women with central hypothyroidism. Therefore, close monitoring of free T4 every 4 to 6 weeks is recommended to allow timely dose adjustments. Hydrocortisone replacement was progressively increased in the third trimester, in accordance with established recommendations for adrenal insufficiency.

To our knowledge, this is one of the few published cases documenting a successful IVF-conceived pregnancy in a woman with germinoma-induced hypopituitarism and thrombotic predisposition, particularly following prior gonadotoxic exposure.

## CONCLUSION

This case underscores the importance of individualized, multidisciplinary care in achieving pregnancy among women with complex endocrine and thrombotic disorders. Comprehensive hormonal replacement, proactive thromboprophylaxis, and tailored ovarian stimulation protocols may enable successful ART outcomes, even in high-risk scenarios.
